# A New Empirical Approach to Intercultural Comparisons of Value Preferences Based on Schwartz’s Theory

**DOI:** 10.3389/fpsyg.2020.01723

**Published:** 2020-07-14

**Authors:** Erich H. Witte, Adrian Stanciu, Klaus Boehnke

**Affiliations:** ^1^Institute of Psychology, University of Hamburg, Hamburg, Germany; ^2^Psychology and Methods, Jacobs University Bremen, Bremen, Germany; ^3^Institute for Gerontology, University of Vechta, Vechta, Germany; ^4^Center for Sociocultural Research, National Research University Higher School of Economics, Moscow, Russia

**Keywords:** values, Schwartz, averaging approach, distribution approach, societal challenges, cross-cultural, European Social Survey

## Abstract

Empirical tests of Schwartz’s theory of culture-level value priorities have predominantly been performed using an averaging approach–as values of the average individual in a culture. However, from a theory of measurement standpoint such an approach seems inadequate. We argue that the averaging approach is an insufficiently accurate methodology in capturing the compatibilities-incompatibilities between values of individuals *within* cultures. We propose an approach based on the distribution of values of individuals in a given culture–the distribution approach. Using data from two rounds of the European Social Survey, we show how frequencies of specific individual value priorities in a culture can be used toward the description of culture-level value preferences. We recommend a re-conceptualization of Schwartz’s culture-level value theory to an orthogonal two-dimensional structure, namely as *Alteration* vs. *Preservation* and *Amenability* vs. *Dominance*, which we explain based on heterogeneity in socioecological indicators across countries. We conclude that societal challenges may influence the cultural value climate across countries.

## Introduction

Culture has recently been defined as a “latent, hypothetical construct [that] cannot be observed directly but can be inferred from its manifestations” (Schwartz, 2014). Yet, the assessment of Schwartz’s values at the culture level has been based on a methodological approach construed with a different definition in mind, namely as “the rich complex of meanings, beliefs, practices, symbols, norms, and values prevalent among people in a society” (Schwartz, 2006). Cultural values are then empirically estimated as values of the average individual in a culture–the averaging approach (Schwartz, 2014). In this article, we raise the point that this method is psychometrically inaccurate.

Averaging or additive methods of assessing a latent construct should be applied only under strict assumptions (Cronbach, 1951; Estes, 1956; McDonald, 1999). In the present case, we know that single value preferences on opposite sides of the value circumplex are meant to correlate negatively and unfold along a two-dimensional structure (see the Supplementary Tables 1, 2). Thus, simply averaging elements with this characteristic cannot be a measure of a latent construct of cultural values. By averaging across individuals (as if they were items measuring a culture’s value preference), one implicitly reduces the data to a unidimensional structure of value priorities. This is typically done in classical scale score calculation for unidimensional scales. The core idea of Schwartz’s individual-level value theory is, however, that–for each and every single value–priorities follow the underlying two-dimensional logic. Adding or averaging scores across individuals discards the conceptual bi-dimensionality (see section “The Status-Quo of Research on Cultural Values”). In our view, a more adequate empirical equivalent of the theory is to base measurement of culture-level values on the frequencies of all individual value preferences in a culture–*the distribution approach* [Gollan and Witte, 2014; for other research applying this reasoning see Kamakura and Mazzon (1991) and Magun et al. (2016)]. The present article sets out to theoretically link the individual and the cultural level by elaborating specifics of empirically determining cultural values under the distribution approach. Please note that our attempt differs from other work that looks at congruency/isomorphism in the value structure between the individual and culture levels while relying on the averaging approach (Fischer et al., 2010; Fischer and Schwartz, 2011).

Furthermore, we discuss how socioecological differences between countries can contribute to the explanation of the here-to-be-specified cultural value dimensions. The secondary goal of this article thus is to provide evidence concerning the added value of the proposed new approach. In doing so, we not only emphasize the methodological shortcomings of the averaging approach, but also show in which ways the results based on the distribution approach distinguish themselves from the results based on the averaging approach. We apply elements of the logic of the convergent and discriminant scale validation and seek to show that socioecological indicators (e.g., socio-economic burdens) explain the positioning of countries along the here-to-be identified culture-level value structure. This exercise will also inform the nomenclature of the novel culture-level dimensions.

## Schwartz’s Theories of Values: Individual and Cultural

The focus of the present work is on the empirical procedure and theory of cultural values. However, because our proposed approach demands a conceptual and empirical link between the theory of individual values and the theory of cultural values, we provide a brief reminder of the core postulates of both theories before we proceed.

### The Theory of Individual Values

Individual values are subjective beliefs of individuals (a) associated with affect, (b) referring to goals that motivate action, (c) transcending specific situations, (d) serving as evaluative standards, (e) ordered according to their *relative* (our emphasis) importance, and (f) guiding individuals’ action (Schwartz, 2012). According to Schwartz, individual values are responses to three universal requirements of human existence, namely needs of people as biological organisms, of agreement in social actions, and of survival and well-being of groups (Schwartz, 1992). Schwartz and colleagues have theorized and shown empirical support for the existence of 10 basic individual values (Schwartz, 1992; Schwartz and Boehnke, 2004). These are: Conformity, Tradition, Security, Power, Achievement, Hedonism, Stimulation, Self-Direction, Universalism, and Benevolence. Since values have specific motivations and goals, the content of any given value is compatible with some and incompatible with others.

The widely accepted representation of individual values is a circumplex model, wherein compatible values border on each other and oppose those which are incompatible. The model simplifies these compatibility-incompatibility relations along a two-dimensional structure, namely Conservation vs. Openness (emphasizing the dichotomy of preservation and change of the *status quo*) and Self-Enhancement vs. Self-Transcendence (emphasizing the dichotomy between personal- and other-related interests). In a recent publication, Schwartz and colleagues have developed a more finely grained value circumplex that entails 19 different value types, which can, however, be collapsed into the ‘classic’ 10 value types (Schwartz et al., 2012). Because representative samples from a sufficient number of countries are until now only available for the 10-value circumplex, we base our research on the ‘classic’ value circumplex.

### The Theory of Cultural Values

Cultural values represent ideals that shape the beliefs and actions of individuals and groups in the culture (Schwartz, 2006). In contrast to individual values, which are in such sense observable constructs that they affect individual behavior, cultural values are abstract constructs (Schwartz, 2014) that manifest in various forms ranging from written artifacts to knowledge that members of the culture communicate amongst each other (Lönnqvist et al., 2012). Assuming the average person to be something like an embodiment of culture, Schwartz proposes cultural values as societal responses to three main challenges to a successful cohabitation (Schwartz, 2006). The type of relations between individuals and groups represents a first challenge, which informs the value-duality of Autonomy (Intellectual and Affective) and Embeddedness. The way individuals act in a responsible manner toward preserving the well-being of their society is a second challenge, which informs the opposing values of Egalitarianism and Hierarchy. And, individuals’ interests regarding the natural and social environment represents a third challenge, which speaks to the contrasting values of Harmony and Mastery. Some empirical support for the–non-orthogonal–three-dimensional structure of these seven cultural values based on the averaging approach has been presented (Schwartz, 2006; Schiefer, 2012). However, the validity of these findings is greatly influenced by the adequacy of the assessment methodology. The current form of Schwartz’s conceptualization of values at the culture level is driven by assuming the existence of a fictitious middle or mean person, a methodological approach that, as we will argue throughout, is inadequately reproducing Schwartz’s very own concept of culture empirically.

## The Status-Quo of Research on Cultural Values

Social science research relies almost exclusively and largely unquestioned on mean scores in drawing conclusions about phenomena at the individual and the culture level (Speelman and McGann, 2013). However, in scale construction, for example, one first demonstrates that items of a scale have sufficient internal consistency (Cronbach, 1951) before using the average score across scale items as a reflection of the measured psychological construct at the individual level. That is, items with low intercorrelations across study participants are discarded to arrive at a meaningful overall indicator of the measured psychological construct.

In research on cultural phenomena, one draws a sample from the population of interest, computes the average score across the sampled individuals, and then interprets the average score as being representative of a psychological construct at the culture level. The procedure uses a fictitious middle individual as the manifestation of a psychological construct at the culture level. The assumption is that the sampled individuals are highly similar amongst each other, or, in statistical terminology, that there is a high positive intercorrelation among the profiles of all sampled individuals. This is problematic, however, because in some situations profiles of individuals can correlate negatively among each other and are unrelated entirely in other cases (Estes, 1956). In cross-cultural research, there is no tradition in demonstrating that individuals within a country have sufficient, so-to-speak, internal consistency. The representation of individual values *in* each culture, in contrast, has a well-supported two-dimensional circumplex structure.

Schwartz’s cultural values are assessed by computing average scores across value-items in each sampled group of participants (Schwartz, 2004). The resulting units of analysis are mean values of samples–in the current case 10 means for the 10 assessed value preferences. In relying on Smallest Space Analysis (SSA), Schwartz has argued that the averaging approach is successful in identifying the aforementioned seven theorized values at the culture level (Schwartz, 2006). SSA, a specific multidimensional scaling (MDS) visualization technique, provides a tool to examine similarities and differences between cases in each set of data, which are interpreted in the proposed model of values at the culture level. We do not question the interpretation of MDS-based results, in particular since the arrival of Confirmatory SSA (Borg et al., 2011). However, we know that culture (as a latent variable) can be approximated by the middle or mean individual in the culture (as manifest variables) *only* in cases where the emergent individual value profiles are correlated across all individuals in a country and if item intercorrelation patterns do not contradict the theoretically proposed and empirically corroborated (Schwartz and Boehnke, 2004) circumplex structure of values at the individual level.

Value profiles of individuals *should* have a specific distribution when one looks at the culture level. Yet, the averaging approach discards the circumplex structure of value priorities at the individual level by reducing the heterogeneity of value profiles to an average one-dimensional score that is in danger of lacking substance in cultures because individuals in their value preferences follow a circumplex, two-dimensional structure. If culture is an abstract construct external to individuals, then, we argue, cultural values are a manifestation of the way values of all individuals in a culture organize collectively.

## The Distribution Approach

Gollan and Witte have developed the distribution approach to the assessment of individual values within a culture by focusing on the exclusion of mean scores from the computation of individual value distribution (Cronbach, 1951; also see Schwartz, 2006; Bilsky, 2008; Strack et al., 2008). They argued that the measurement of individual value preferences on an ordinal scale eliminates individual means *a priori* as they have no intercept (zero point) and this kind of elimination is generally proposed by Schwartz. The authors showed that the two-dimensional circumplex structure of value priorities at the individual level can be reliably unfolded as rank orders of value priorities–value priorities of individuals can be organized in an orderly fashion from highest prioritized to lowest prioritized (Gollan and Witte, 2014). To give a brief example already here (see also Table 1): According to Schwartz’s individual-level value theory, individuals who most strongly prefer Stimulation values (Rank 1) should (see also Line 7, Stimulation’, in Table 1) as well have relatively high preferences for Hedonism and Self-Direction values, placed adjacent to Stimulation values in the circumplex (Ranks 2/3, expected rank: 2.5). Achievement and Universalism values should follow in their level of preference (Rank 4.5). Power and Benevolence values would be next (Rank 6.5), Tradition, Conformity, and Security values can then be expected to receive the lowest preference ratings (Ranks 8/9/10, expected rank 9).

**TABLE 1:** Ideal value types based on the rank order of values.

	Rank order of individual values									
Ideal value type	CO	TR	SE	PO	AC	HE	ST	SD	UN	BE
Conformity	1	2	3.5	5.5	7.5	9.5	9.5	7.5	5.5	3.5
Tradition	2	1	3.5	5.5	7.5	9.5	9.5	7.5	5.5	3.5
Security	3	3	1	3	5.5	7.5	9.5	9.5	7.5	5.5
Power	5	5	2.5	1	2.5	5	7.5	9.5	9.5	7.5
Achievement	7	7	4.5	2.5	1	2.5	4.5	7	9.5	9.5
Hedonism	9	9	6.5	4.5	2.5	1	2.5	4.5	6.5	9
Stimulation	9	9	9	6.5	4.5	2.5	1	2.5	4.5	6.5
Self-Direction	7	7	9.5	9.5	7	4.5	2.5	1	2.5	4.5
Universalism	5	5	7.5	9.5	9.5	7.5	5	2.5	1	2.5
Benevolence	3	3	5.5	7.5	9.5	9.5	7.5	5.5	3	1

*The main diagonal is not to be confused with the main diagonal in a table of intercorrelations; numbers in every line correspond to value rank-orders which are based on value proximities among sectors of the circumplex model proposed by Shalom H. Schwartz; the column ‘Ideal Value Types’ provides the label for the row specified rank orders of the 10 value types – each row corresponds to the theorized rank order of a specific value type; number ‘1’ is the starting value type of a theorized rank order; CO = Conformity; TR = Tradition; SE = Security; PO = Power; AC = Achievement; HE = Hedonism; ST = Stimulation; SD = Self-Direction; UN = Universalism; BE = Benevolence.*

The logic of eliminating mean scores (through ranking) should likewise be utilized in intercultural comparative research, since this would liberate research from interval scale restrictions and provide additional control in testing theoretical propositions in a valid way. The distribution approach satisfies the conditions of a valid psychometric score because (i) it maintains the two-dimensional structure of value priorities and (ii) uses the ordinal scale, which is more robust than the interval scale in accounting for cross-cultural variations in meaning of expression.

**FIGURE 1:**
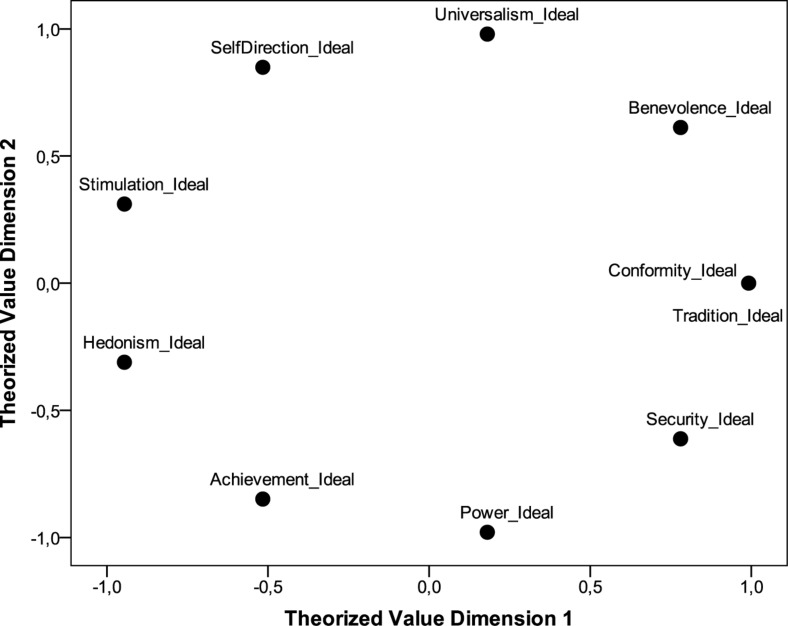
Circumplex model of basic value priorities as reproduced in the distribution approach.

We propose a theory-driven procedure to arriving at culture-level value priorities from individual-level value priorities. The circumplex model of individual-level value preferences is unfolded as a matrix of ideal value types, namely, individual value priorities are organized according to their proximity in the theorized sectors of the two-dimensional space. Sectors with the same distance receive the same rank (see Table 1). In the matrix, which is not to be confused with a full correlation matrix, the highest value preference (of an individual) has the highest rank (i.e., ‘1’). All other ranks follow as depending on their distance from the sector of the initial rank. Ties are used for equal distances. Intercorrelations of these ideal value types are the empirical reproduction of the theorized circumplex model (see Table 2; also see Figure 1). The classification of an individual into an ideal value type is done by correlating these ideal value types with the measured value profiles of study participants (from the dataset). This classification is valid only if the individuals’ value preference profile correlates to at least *r* ≥ 0.50 with at least one ideal-type rank order because only then the surveyed individuals follow the theoretical assumptions of the circumplex model (Borg et al., 2015).

**TABLE 2:** The unfolding of the circumplex model of individual values as inter-correlations among ideal value types.

	Ideal value type	1	2	3	4	5	6	7	8	9	10
1	Conformity	1									
2	Tradition	0.99	1								
3	Security	0.76	0.76	1							
4	Power	0.17	0.17	0.74	1						
5	Achievement	−0.50	−0.50	0.11	0.73	1					
6	Hedonism	−0.94	−0.94	−0.54	0.13	0.75	1				
7	Stimulation	−0.94	−0.94	−0.93	−0.47	0.22	0.79	1			
8	Self-Direction	−0.50	−0.50	−0.93	−0.93	−0.44	0.22	0.75	1		
9	Universalism	0.17	0.17	−0.45	−0.93	−0.93	−0.47	0.13	0.73	1	
10	Benevolence	0.76	0.76	0.23	−0.45	−0.93	−0.93	−0.54	0.11	0.74	1

*Intercorrelations are calculated on the value rank orders presented in Table 1: each ideal value type (each row) of Table 1 is row-wise correlated with *all* (including itself) ideal value types (all rows).*

Eleven value classes are possible: the 10 ideal value types as proposed by Schwartz and a non-classified type which includes cases that do not meet the *r* ≥ 0.50 threshold. By using the less restrictive ordinal measurements, it is possible then to rank-order the distribution of value classes in a culture according to frequencies. The value type rank orders are subsequently used to identify cross-cultural similarities and differences. Countries with equal frequency distributions across all value types are similar cultures. The procedure is fully described in the section “Results.” Furthermore, Table 3 provides a step-by-step clarification of the similarities and differences between the methods currently used and the one we propose to measure value theories as proposed by Schwartz.

**TABLE 3:** Existent methods and the here-proposed one in measuring the two value theories proposed by Shalom H. Schwartz.

Step	Individual level theory	Culture level theory	
		Distribution approach	Averaging approach
1	Each item on the PVQ-21 instrument corresponds to 1 and only 1 of the 10 theorized individual level value types		
2	Participants answer the PVQ-21 instrument		
3	MRAT correction is calculated by averaging across all 21 items - subtraction of the individual mean		
4	Index for each of the 10 theorized value types is constructed by averaging across the appropriate PVQ items for each individual		The mean rating for each of the 21 items is computed for each country
5	MRAT correction is applied to the indices constructed in Step 4		
6		The circumplex model of individual value typologies is unfolded empirically into 10 ideal value types based on the empirical results found in Schwartz (1992)	Mean ratings calculated in Step 4 are correlated across countries and other cultural groups
7		Fit between the ideal value types in Step 6 and the corrected indices in Step 5 is calculated as a correlation coefficient	The mean importance rating of the value items found in Step 6 is analyzed in a confirmatory multidimensional scaling analysis. This corresponds to the approach pursued for individual-level values; a universal two-dimensional circumplex structure of seven culture-level values is proposed.
8		A person’s classification into a value class is made according to the correlation coefficient in Step 7: a. Correlation threshold is *r* = 0.50 b. Highest correlation coefficient defines the value class of a person c. 11 value classes are possible: 10 theorized, 1 non-classified (*r* < 0.50)	Mean scores of culture-level value orientation for each cultural group are used to compute the absolute differences between the cultural groups as an index of the value profile similarity between groups.
9		Frequency scores for each value classes in each country is calculated	
10		Frequency scores in Step 9 are transformed into rank order types	
11		Principal component analysis extracts factor scores and saves factor loadings based on rank order types in Step 10	
12		Factor scores in Step 11 represent the common value space across all countries included in the analysis	
13		Factor loadings in Step 11 represent a country’s unique similarity with the common value space in Step 12	

Elaborations presented in Table 3 clarify that both Schwartz’s averaging approach to determining culture-level values and our to-be-developed distribution approach use ipsatization of raw value preference scores, the so-called MRAT correction. In recent contributions, this has been criticized (He and Van De Vijver, 2015; Borg and Bardi, 2016; He et al., 2017). It has convincingly been shown that a person’s average rating across all values has a substantive meaning and is not just an indication of a merely technical response tendency to mark survey items high or low. Nevertheless, we follow Schwartz (Schwartz, 2009, 2020), who ascertains that when relating value preferences to other variables, the appropriateness of ipsatizing depends on one’s aims and assumptions. He points out that in cases, where it is not a single value that is of interest to a researcher, but the entire set of values around the circumplex, ipsatizing is important, because values relate to other variables both through facilitating and inhibiting processes. At a minimum, relations depend on the balance between two opposing values. More often, multiple opposing values may be involved. For Schwartz, the trade-off between opposing values is of crucial relevance, and a trade-off that requires considering the *relative* importance of the values involved for each individual. Ipsatizing converts the absolute importance of the value scores into relative importance scores. We find this argumentation convincing for our case as well. Our approach’s very purpose is to consider all values simultaneously and in their culture-specific distribution.

## Socioecological Explanations to Cultural Differences and Similarities

We propose a socioecological approach (Oishi and Graham, 2010; Oishi, 2014) to understanding values and their diffusion at the cultural level. Socioecological explanations have been applied in empirical tests of all main cultural values theories, including Schwartz’s, Hofstede’s, Huntington’s, and Inglehart’s (Georgas et al., 2004). Socioecological explanations situate human psychology in context and indicate that psychological factors are reactions of individuals coming in contact with the respective objective reality that prevails in their contexts of living (Oishi and Graham, 2010; Oishi, 2014). As a matter of fact, Shalom Schwartz has theorized that values at the cultural level represent ways in which the collective of individuals deal with challenges of living together in a shared space of interaction, preserving the social structure, and acting in the interest of the natural and social environment (Schwartz, 2006). He theorized this after having similarly suggested that values of individual members of a culture are grounded in three basic human needs, namely, biological, coordinated social action and survival and welfare of the group (Schwartz, 2012). Schwartz resorted to a socioecological explanation whereby he correlated distal societal indices (objective indicators; e.g., Democracy index, Household size, Gross National Income per capita) with the found culture-level value dimensions and interpreted the moderate to high coefficients as support for his conceptualization. More recently, he argued that values at the culture level draw heavily from distal factors such as ecology and the history of a country which are thereby transmitted and imprinted into individuals’ personal values (Schwartz, 2014).

In following this tradition, the here-to-be-identified culture-level value dimensions must also be situated in the objective reality wherein individuals reside. In this article, we look at objective indicators of the economic and socio-political structures across societies and how they inform the cultural value dimensions. The economic sphere is assessed via the Gross Domestic Product per capita index (GDPpc), as countries’ overall economic status, and the GINI index, as countries’ level of income inequality. The socio-political sphere is assessed via literacy rate (years of education), general societal state of health (life expectancy as published by the Human Development Program), degree of cultural and ethnic diversity (ethnic fractionalization) (Alesina et al., 2003), civil liberties (Freedom House), percentage of religious people, and percentage of people not classifiable into one of the 10 basic value types in the preparatory classification analysis described below. We see the latter as people who exhibit an arbitrarily erratic structure of value preferences. We will attend to the meaning of this variable again later.

Moreover, we attempt to provide first steps toward a conceptual redefinition of culture-level values. We argue that the way values of individuals organize at the cultural level is a manifestation of perceived worries across societies. According to Boehnke and colleagues, worries refer to an emotionally disturbing cognitive mind set of individuals that the state of an object (micro or macro) may suffer alterations from the preferred state (Boehnke et al., 1989). Worries can concern the economic sector, for example, many people in a country living in poverty, or the socio-political sector, for example, conflicts among varying groups in a country (Boehnke et al., 1998). Indeed, values are none other but innate goals of individuals (Schwartz, 2012) which may be at risk of unfulfillment when the object of the goal is perceived as being in an undesirable state (Schwartz et al., 2000). We argue that specific cultural values will thrive across socioecological contexts because individuals perceive their environments accordingly as posing obstacles or facilitating their well-being (Diener et al., 2013; Oishi and Diener, 2014). We examine associations between the to-be-developed culture-level value dimensions and subjective well-being (SWB). The basic assumption here is that in a country there is a single peaked distribution and reactions could be measured on an interval-like scale. We do not have specific expectations as to how these predictions will unfold, since our work seeks to explore new methodological venues of the theory and is not meant to further confirm its validity.

## Materials and Methods

### Data

Data from Rounds 6 and 7 of the European Social Survey (ESS) were used (European Social Survey, 2012, 2014). Twenty countries were available in both rounds, namely: Belgium (BE), Switzerland (CH), Czechia (CZ), Germany (DE), Denmark (DK), Estonia (EE), Spain (ES), Finland (FI), France (FR), Great-Britain (GB), Hungary (HU), Israel (IL), Ireland (IE), Lithuania (LT), the Netherlands (NL), Norway (NO), Poland (PL), Portugal (PT), Sweden (SE), and Slovenia (SI). Nine countries were available only in Round 6 of the ESS, namely: Albania (AL), Bulgaria (BG), Cyprus (CY), Iceland (IS), Italy (IT), Russian Federation (RU), Slovakia (SK), Ukraine (UA), and Kosovo (XK). One country was only available in Round 7 of the ESS, namely Austria (AT). These represent our country-units of analysis: there were 30 country-units of analysis, out of which 20 were available from both ESS rounds, 9 from ESS round 6, and 1 from ESS round 7. Thus, the data set comprised 60 country-units from two ESS rounds.

Listwise deletion of missing data was enacted: Single cases were discarded from analyses if they lacked information on one or more of the 10 individual value preferences; this was the case for *n*_*ESS6*_ = 988 and *n*_*ESS7*_ = 1,101 cases in the two waves, respectively. Subsequently, cases were discarded if response scores were identical across all 10 individual value preferences (*n*_*ESS6*_ = 244 and *n*_*ESS7*_ = 110). The final data sets encompassed *N*_*ESS6*_ = 53,441 and *N*_*ESS7*_ = 38,974 cases. The European Social Survey utilizes national random probability samples. To further augment country representativeness, the provided design and population weights were applied for the current analyses. All the study materials (guide included) are deposited online and are open access (Witte et al., 2019).

### Measures

#### Individual Values

*Individual values* were assessed using a 21-item version of the Portrait Value Questionnaire (PVQ-21) (Schwartz, 2003). Each item presents a brief description of some person and pertains to one specific value type. Of the 10 values, nine are assessed by two items each, Universalism is assessed by three items. Respondents were asked to indicate to what degree a fictitious person was like themselves on a response scale ranging from 1 (very much like me) to 6 (not like me at all). An item example–for Achievement values–is: “It is important to her [him] to show her [his] abilities. She [He] wants people to admire what she [he] does.” For each case, individual values were calculated by averaging responses on the–two to three–value-specific items to construct the value profile for each subject. Subsequently, we performed the so-called MRAT correction (see above), as prescribed by Schwartz for ESS data (Boehnke et al., 1989). We, thus, work with scale scores as manifest variables, for which reliability and validity has been confirmed in a voluminous body of prior work.

#### Economic Indicators

*The Gross Domestic Product* per capita 2015 (GDPpc) is a yearly nation-level indicator of all goods and services produced in a nation, estimated in US $, relative to the nation’s population size (The World Bank, 2015). The world countries rank was used; countries are ranked from highest to lowest in terms of goods and services. The *Gini 2012* coefficient is a nation-level indicator that indexes the amount of income inequality among a nation’s residents (The World Bank, 2012). Higher scores indicate more inequality.

#### Socio-Political Indicators

*Life expectancy* (at birth) 2015 is a nation-level indicator of the number of years a newborn infant could expect to live if the age-specific mortality rate at the time of birth remains unchanged (UNDP, 2015). Higher values on the index signify higher life expectancy. *Education* was assessed as completed years of full-time education. *Ethnic fractionalization* is a nation-level indicator of the probability that two members of the same country do not belong to the same ethno-linguistic group (Alesina et al., 2003). Higher values on the indicator signify higher ethnic diversity. Freedom House’s index of *Civil Liberty 2017* is a nation-level indicator of civil liberties. Higher scores on the index signify *fewer* civil liberties (Freedom House, 2017). *Proportion of religious people* was assessed as the proportion of individuals who declare belonging to a religious denomination.

#### Subjective Well-Being

*Subjective well-being* was assessed as life satisfaction, the cognitive component of well-being (Pavot and Diener, 2008), via the ESS-available single item, “All things considered, how satisfied are you with your life as a whole nowadays?” The response options ranged from 0 (extremely dissatisfied) to 10 (extremely satisfied).

### Procedure

A first step was to classify each individual in the data set according to their fit to the circumplex model, which followed a continuous progression from ‘imperfect fit’ to ‘perfect fit’ [for the actual procedure and results, see repository materials (Witte et al., 2019)]. Fit was statistically assessed as Pearson’s product-moment correlations [identical to Spearman-rank-correlation (Bortz et al., 2008)] between individuals’ value profiles (the measured 10 values of each case in the data set) and ideal value profiles (the theorized rank-ordered 10 value types, see Table 1, read line-wise). The individuals’ value profile was that of the 10 value types proposed by Schwartz, obtained from ipsatized ESS data. The ideal value types were assessed on the ordinal level and were taken from Table 1. Alternatively, one could first have transformed the value score profile into a value rank order. However, we decided against this procedure because of the mathematical reduction that emerges regarding ties (e.g., 1.5). In both rounds of the ESS, the correlation of the classifications by ipsatized preference scores and by ranks was *r* = 0.79, which provided further support for the ranking procedure based on raw scores.

For each individual case in the dataset, 10 correlation coefficients resulted. To illustrate: The raw value profile of a case was first correlated with the ideal value profile of Conformity (highest score for Conformity), second with the ideal profile of Tradition (highest score for Tradition), third with the ideal profile of Security (highest score for Security), and so on. Correlation coefficients stronger than *r* = 0.50 (threshold) classified cases into 1 value type. For cases with *more* than one correlation coefficient above the threshold, the *strongest* coefficient defined the value class of the case. Cases with no correlation coefficient above the threshold were *not* classified into one of the 10 value categories but into a new category–the non-classified.

Next, for each *country-unit* in the data set, frequencies of people classified as ‘belonging’ to a particular value class were calculated. The more frequent the classification of people into a value type in a country-unit, the higher the priority of the respective value class in the culture. Conversely, the less frequent the classification of people into a value type in a country-unit, the lower the priority of the respective value in the culture. Based on standardized frequency scores (percentages), value classifications in a culture were then rank-ordered from highest priority to lowest priority.

Subsequently, we examined the dimensionality of cultural values. We analyzed the country-units in the data set according to their similarities and differences on the ranked percentages of value classes. Formally, the 11 value classifications were treated as cases (rows in the dataset) and value rank orders per country-unit were treated as variables (columns in the dataset) in a Principal Component Analysis (PCA). We correlated the country-units over the ranks of the value types due to their percentages. We chose PCA as a variant of exploratory factor analysis although traditional rules of thumb pertaining to the ratio of cases (n rankings of value preferences according to the distribution approach, here 11) to variables (p country-units, here 60) were not satisfied. In a simulation study, Preacher and MacCallum showed that this rarely has decisive consequences for the results if only few components are extracted from the covariance matrix (Preacher and MacCallum, 2002). Important to note, the aim of this analysis is to find latent variables that allow us to group countries.

The emergent *factor scores* (not loadings!) of each factor are orthogonal and reflect the best fitting distribution of value priorities for all country-units. In other words, the emergent factor scores are to be interpreted as the structure of the latent cultural value profiles in Europe. If more than one factor exists, the observed empirical value profile of a country-unit is a mixture of more than one latent profile that weights the extracted factor scores (pertinent to all country-units) by the unique *loadings* of the respective country-unit.

In more formal terms, factor loadings indicate the similarities of each country-unit to the latent value profile carried by the profile of each set of factor scores. Similarity in cultural values of two country-units is indicated by similar factor loadings on the common latent profile (the factor scores). For illustration purposes, the observed cultural value profile in a country-unit is given by the following formula:

(1) $$Y_{C}^{V}=l_{F\,1}^{V}\,f_{F\,1}^{V}+l_{F\,2}^{V}\,f_{f\,2}^{V}+\ldots \,\ldots$$

In Formula 1, Y is the observed rank-ordered value profile *V* in Culture *C*, l is the country-unit loading on Factor 1 (indicated by subscript *F*1) and on Factor 2 (indicated by subscript *F*2), and f is the hidden value profile on Factor 1 (indicated by subscript *F*1) and on Factor Score 2 (indicated by subscript *F*2). Factor loadings are the varimax rotated (maximizes the variance of the loadings on the orthogonal factors) factor solutions of the PCA per country-unit and Factor Scores ‘1’ and ‘2’ are constant for all country-units (the *z*-transformed value profiles across country-units). If one uses SPSS as statistical software (other software will provide identical coefficients), one finds factor loadings in the output and the factor scores as new variables in the data set, provided that one ‘saves’ the PCA results as regression coefficients.

In other words, the emergent factors with their scores of values can be interpreted as the latent profile of cultural values in Europe that can be used to predict the observed profile of each country. Similarity in cultural values of two countries is indicated by similar factor loadings because the common latent profile (the factor scores) is used with equal weights in both countries. The factor scores are independent elements that predict the observed (empirical) value profile of a country. The factor scores are, due to the orthogonal rotation, uncorrelated and have nothing to do with the factor loadings. Factor loadings can be different for two countries because they can have unique weights on the common factor scores. Correlations between loadings imply that the *unique* weights across countries are similar. Factor scores, however, are always independent. There is no redundancy if loadings are correlated because the factor scores (the latent cultural value profiles in Europe) cannot be predicted from factor loadings (the latent cultural value profile of a specific country).

## Results

### Value Type Classification and Rank Order Within Cultures

Table 4 documents the classification of participants into value types and the rank order of these value classes across all countries (country-units) in Round 6 and 7 of the ESS. Finland was the country with the highest percentage of people preferring Universalism (32.0 %) as their ‘first choice,’ so-to-speak, and the Russian Federation was the country with the highest percentage of people preferring Power (1.9 %), values that were typically the most and least preferred in the European context. Vice-versa, Lithuania was the country with the lowest percentage of people preferring Universalism (2.0 %) and, for example, Germany was among the countries that had the lowest percentage of people preferring Power (0.1 %). The percentage of unclassifiable participants was highest in Israel (54.0 %) and lowest in Sweden (18.7 %), meaning that these countries had the highest and the lowest percentage of people with value preferences that did not fit the individual-level value circumplex.

**TABLE 4:** Proportions of classified individuals into each value-type and their rank order across countries in rounds 6 and 7 of the ESS.

Country code	Round ESS	CO	TR	SE	PO	AC	HE	ST	SD	UN	BE	NON- CLASS	*TOTAL N*
AL	6	10.3 (3)	6.9 (5)	5.9 (6)	1.7 (9)	0.2 (11)	1.2 (10)	1.6 (8)	4.1 (7)	8.4 (4)	12.5 (2)	47.2 (1)	1104
AT	7	8.5 (4)	4.6 (6)	2.2 (8)	0.4 (10)	0.3 (11)	1.9 (9)	4.5 (7)	8.0 (5)	13.3 (3)	17.5 (2)	38.7 (1)	1793
BE	6	5.1 (6)	4.2 (7)	1.1 (8)	0.2 (10)	0.2 (11)	1.0 (9)	5.1 (5)	11.0 (4)	20.0 (2)	19.7 (3)	32.4 (1)	1860
	7	5.8 (5)	3.8 (6)	1.6 (8)	0.3 (10)	0.3 (11)	1.3 (9)	3.2 (7)	11.5 (4)	20.5 (2)	18.7 (3)	32.9 (1)	1767
BG	6	22.5 (2)	13.3 (3)	3.6 (5)	0.5 (11)	0.6 (10)	3.3 (6)	2.9 (7)	2.1 (9)	2.6 (8)	11.7 (4)	36.8 (1)	2171
CH	6	4.0 (6)	3.2 (7)	1.3 (9)	0.4 (10)	0.4 (10)	1.5 (8)	5.9 (5)	11.9 (4)	21.1 (2)	14.6 (3)	35.7 (1)	1483
	7	3.7 (6)	3.4 (7)	0.7 (9)	0.3 (10)	0.1 (11)	1.6 (8)	5.5 (5)	14.0 (4)	21.3 (2)	15.0 (3)	34.5 (1)	1517
CY	6	4.6 (6)	9.5 (4)	2.5 (9)	0.3 (11)	0.5 (10)	3.3 (7)	5.5 (5)	2.5 (8)	10.5 (3)	22.0 (2)	38.7 (1)	1109
CZ	6	11.5 (3)	7.3 (5)	3.7 (8)	0.9 (11)	1.2 (10)	7.8 (4)	4.5 (7)	3.1 (9)	6.2 (6)	16.6 (2)	37.2 (1)	1947
	7	10.8 (3)	6.7 (5)	5.1 (8)	1.2 (11)	2.0 (10)	6.1 (7)	6.9 (4)	3.6 (9)	6.4 (6)	14.9 (2)	36.2 (1)	1845
DE	6	3.8 (6)	2.9 (7)	1.1 (8)	0.2 (10)	0.2 (11)	1.0 (9)	5.6 (5)	11.6 (4)	20.7 (2)	19.1 (3)	33.7 (1)	2933
	7	3.4 (6)	2.4 (7)	0.7 (8)	0.1 (11)	0.3 (10)	0.6 (9)	3.9 (5)	12.1 (4)	27.7 (2)	20.2 (3)	28.6 (1)	3006
DK	6	4.5 (6)	0.7 (8)	0.6 (9)	0.3 (10)	0.2 (11)	1.2 (7)	7.2 (5)	16.7 (4)	24.1 (2)	18.3 (3)	26.2 (1)	1621
	7	4.7 (6)	1.0 (7)	0.9 (8)	0.2 (10)	0.1 (11)	0.8 (9)	5.9 (5)	15.8 (4)	22.8 (2)	17.3 (3)	30.3 (1)	1483
EE	6	8.9 (4)	4.2 (6)	1.9 (9)	0.2 (11)	0.3 (10)	2.6 (8)	3.5 (7)	7.0 (5)	17.4 (3)	25.7 (2)	28.1 (1)	2343
	7	10.4 (4)	5.9 (6)	1.6 (9)	0.4 (10)	0.0 (11)	2.1 (8)	4.3 (7)	6.8 (5)	15.9 (3)	26.0 (2)	26.5 (1)	2033
ES	6	6.8 (5)	5.8 (6)	0.6 (9)	0.2 (11)	0.3 (10)	0.6 (8)	1.6 (7)	7.6 (4)	24.1 (2)	28.6 (1)	23.7 (3)	1869
	7	8.2 (4)	4.5 (6)	0.7 (8)	0.2 (10)	0.1 (11)	0.6 (9)	1.4 (7)	8.0 (5)	23.1 (3)	28.5 (1)	24.7 (2)	1904
FI	6	3.8 (6)	1.1 (7)	0.8 (9)	0.1 (11)	0.3 (10)	1.1 (8)	4.4 (5)	12.5 (4)	28.4 (1)	26.4 (2)	21.2 (3)	2156
	7	3.3 (6)	0.9 (7)	0.6 (8)	0.2 (10)	0.1 (11)	0.6 (8)	3.9 (5)	12.1 (4)	32.0 (1)	24.9 (2)	21.3 (3)	2049
FR	6	3.2 (5)	2.6 (7)	0.9 (9)	0.3 (11)	0.3 (10)	1.3 (8)	3.1 (6)	10.0 (4)	27.6 (2)	19.2 (3)	31.5 (1)	1959
	7	3.4 (5)	2.5 (6)	1.0 (9)	0.1 (10)	0.1 (11)	1.0 (8)	2.1 (7)	11.7 (4)	27.2 (2)	19.3 (3)	31.6 (1)	1901
GB	6	8.4 (4)	3.9 (6)	1.4 (8)	0.7 (10)	0.3 (11)	1.4 (9)	3.6 (7)	7.8 (5)	16.2 (3)	25.0 (2)	31.3 (1)	2254
	7	6.1 (5)	3.1 (6)	1.3 (8)	0.3 (11)	0.4 (10)	1.1 (9)	3.1 (7)	7.6 (4)	21.8 (3)	24.7 (2)	30.5 (1)	2222
HU	6	7.4 (4)	6.2 (5)	3.2 (9)	0.9 (10)	0.8 (11)	5.7 (6)	3.9 (7)	3.8 (8)	7.7 (3)	10.2 (2)	50.3 (1)	1963
	7	5.4 (5)	6.7 (3)	4.5 (7)	0.7 (10)	0.6 (11)	4.8 (6)	3.6 (9)	4.1 (8)	6.2 (4)	10.4 (2)	53.0 (1)	1513
IE	6	9.0 (4)	5.2 (7)	1.0 (9)	0.2 (11)	0.2 (10)	2.2 (8)	5.8 (6)	6.5 (5)	13.0 (3)	21.4 (2)	35.4 (1)	2601
	7	7.6 (4)	5.6 (6)	1.7 (9)	0.5 (11)	0.8 (10)	2.1 (8)	4.9 (7)	6.5 (5)	12.2 (3)	19.7 (2)	38.3 (1)	2379
IL	6	7.7 (3)	7.1 (4)	3.7 (8)	1.2 (11)	1.7 (10)	2.8 (9)	4.0 (6)	4.0 (7)	5.6 (5)	8.2 (2)	54.0 (1)	2338
	7	7.7 (5)	8.0 (4)	3.2 (8)	1.0 (10)	0.9 (11)	2.7 (9)	3.5 (7)	5.6 (6)	8.1 (3)	11.0 (2)	48.2 (1)	2323
IS	6	2.4 (6)	1.1 (7)	0.5 (9)	0.1 (10)	0.1 (10)	0.7 (8)	8.4 (5)	15.5 (4)	28.2 (1)	21.1 (3)	21.8 (2)	739
IT	6	7.6 (5)	8.4 (4)	1.9 (9)	0.4 (10)	0.1 (11)	2.1 (8)	3.0 (7)	5.6 (6)	13.0 (3)	27.7 (2)	30.2 (1)	905
LT	6	8.8 (4)	14.1 (2)	6.8 (5)	1.7 (10)	2.9 (8)	9.5 (3)	4.3 (7)	1.3 (11)	2.2 (9)	5.4 (6)	43.0 (1)	2098
	7	11.2 (3)	12.4 (2)	8.5 (4)	1.4 (11)	2.1 (8)	8.4 (5)	4.3 (7)	1.5 (10)	2.0 (9)	6.9 (6)	41.3 (1)	2222
NL	6	3.9 (6)	1.7 (8)	0.6 (10)	0.6 (9)	0.3 (11)	1.7 (7)	7.3 (5)	15.3 (4)	25.7 (2)	16.3 (3)	26.7 (1)	1825
	7	4.6 (6)	1.9 (7)	0.8 (9)	0.2 (11)	0.4 (10)	1.8 (8)	6.7 (5)	15.6 (4)	23.3 (2)	16.0 (3)	28.7 (1)	1821
NO	6	8.1 (5)	1.3 (7)	0.9 (9)	0.2 (10)	0.2 (11)	1.1 (8)	5.0 (6)	13.2 (4)	24.3 (1)	23.0 (2)	22.7 (3)	1608
	7	9.1 (5)	1.3 (7)	0.8 (8)	0.4 (10)	0.3 (11)	0.8 (8)	4.1 (6)	11.5 (4)	23.6 (2)	25.0 (1)	23.2 (3)	1422
PL	6	19.1 (3)	8.1 (5)	2.5 (8)	0.5 (10)	0.5 (11)	1.7 (9)	2.6 (7)	3.0 (6)	8.1 (4)	28.0 (1)	26.0 (2)	1859
	7	19.9 (3)	11.7 (4)	1.9 (8)	0.2 (11)	0.2 (10)	0.8 (9)	2.4 (7)	2.8 (6)	7.8 (5)	26.5 (1)	25.8 (2)	1584
PT	6	9.5 (4)	9.9 (3)	2.0 (9)	0.5 (10)	0.2 (11)	2.2 (8)	2.3 (7)	4.4 (6)	7.8 (5)	14.9 (2)	46.4 (1)	2129
	7	7.4 (5)	6.1 (6)	2.2 (8)	0.2 (11)	0.2 (10)	1.0 (9)	3.2 (7)	8.9 (4)	17.0 (3)	17.1 (2)	36.6 (1)	1240
RU	6	11.9 (3)	10.0 (4)	5.2 (5)	1.9 (9)	1.6 (11)	4.2 (6)	3.3 (8)	1.8 (10)	3.6 (7)	13.0 (2)	43.3 (1)	2398
SE	6	3.6 (6)	1.0 (7)	0.5 (9)	0.4 (10)	0.1 (11)	0.8 (8)	5.9 (5)	16.1 (4)	29.0 (1)	19.8 (3)	22.7 (2)	1834
	7	2.7 (6)	1.6 (7)	0.4 (9)	0.1 (10)	0.1 (10)	1.0 (8)	4.8 (5)	18.2 (4)	31.4 (1)	20.9 (2)	18.7 (3)	1761
SI	6	8.5 (5)	9.0 (4)	2.0 (9)	0.4 (10)	0.2 (11)	2.1 (8)	4.3 (7)	5.6 (6)	11.4 (3)	15.5 (2)	40.9 (1)	1242
	7	7.3 (5)	7.1 (6)	1.7 (8)	0.1 (11)	0.4 (10)	1.3 (9)	3.8 (7)	9.8 (4)	12.4 (3)	16.1 (2)	40.0 (1)	1189
SK	6	17.1 (3)	12.1 (4)	2.9 (8)	0.5 (11)	1.8 (10)	5.9 (5)	4.0 (7)	1.8 (9)	4.7 (6)	18.9 (2)	30.2 (1)	1817
UA	6	13.5 (3)	7.9 (4)	6.2 (5)	1.6 (11)	1.8 (9)	4.6 (6)	2.9 (8)	1.8 (10)	3.6 (7)	14.3 (2)	41.9 (1)	2062
XK	6	17.1 (2)	11.4 (4)	4.7 (5)	0.6 (11)	1.1 (9)	2.4 (7)	0.9 (10)	2.1 (8)	3.3 (6)	14.3 (3)	42.0 (1)	1214

*Columns and rows must be rotated (columns become rows) to arrive at the data framework that was used in the principal component extraction. This presentation is for a better print output only. Proportions are rounded to the first decimal; parentheses define value rank order; CO = Conformity; TR = Traditionalism; SE = Security; PO = Power; AC = Achievement; HE = Hedonism; ST = Stimulation; SD = Self-Direction; UN = Universalism; BE = Benevolence; NON-CLASS = Non-classified; AL = Albania; AT = Austria; BE = Belgium; BG = Bulgaria; CH = Switzerland; CY = Cyprus; CZ = Czechia; DE = Germany; DK = Denmark; EE = Estonia; ES = Spain; FI = Finland; FR = France; GB = United Kingdom; HU = Hungary; IE = Ireland; IL = Israel; IS = Iceland; IT = Italy; LT = Lithuania; NL = the Netherlands; NO = Norway; PL = Poland; PT = Portugal; RU = Russian Federation; SE = Sweden; SI = Slovenia; SK = Slovakia; UA = Ukraine; XK = Kosovo.*

Overall, about 30 % of the participants could not be classified into one of the value types theorized by Shalom Schwartz, as the value preferences of these cases were diffuse and non-systematically ordered. In our analyses, we took this quantity into account and used it as a variable. For Round 6, the median value classification was Benevolence (19.50 % of N) and the modal value classification was Non-classified (34.70 % of N), and for Round 7 once again the median value classification was Benevolence (21.50 % of N) and the modal value classification was Non-classified (30.10 % of N).

### Structure and Meaning of Cultural Values as Informed by the Distribution Approach

A PCA with varimax rotation was conducted using–60–country-units as “variables” and ranks of percentages of most preferred values (plus percentage of unclassifiable individuals) as–11–cases [or items/columns, for procedure and output, see repository materials (Witte et al., 2019)]. Data of countries with measurements on both rounds allowed us to cross-validate the cross-cultural structure of values. Value rank orders of the 10 countries in only one round of the ESS were assumed as robust for the round without observed data. Effectively each country thus had two columns in the analyzed data set, regardless of whether it had seen one or two rounds of ESS surveying (see Table 3). This was done to avoid having results biased toward the countries with original data in the two rounds. This assumption was justified by the high correlations of countries in the two rounds.

The PCA extracted three factors with eigenvalues above 1. However, the scree plot indicated that two factors explained almost all the overall variance (94.88%). Subsequently, we conducted a parallel analysis (O’Connor, 2000; Hayton et al., 2004) whereby we extracted principal components from a randomly generated dataset with the same specification as ours. The procedure recommends the retention of factors with eigenvalues greater than 1 that are also greater than the eigenvalues of factors from the randomly generated data set. The results of the parallel analysis showed that a two-factor solution was only slightly worse than a single factor solution [see repository output (Witte et al., 2019)]. Based on all these criteria, we decided that two factors were most reliable in summarizing the data at hand. After rotation, these factors explained the following proportions of total variance, Factor 1, *σ*^2^ = 60.71 % (eigenvalue of 48.90), and Factor 2, *σ*^2^ = 34.17 % (eigenvalue of 8.03) [for factor loadings see the Supplementary Table 1; for factor eigenvalues and factor retention output, see repository materials (Witte et al., 2019)].

As shown in Table 5, Factor 1 was represented by the following rank order of value typologies: Universalism, Self-Direction, Benevolence, and Stimulation vs. Achievement, Security, Power, Hedonism, Tradition, and Conformity. Factor 2, on the other hand, was represented by this rank order of value typologies: Conformity, Tradition, Benevolence, Security, and Hedonism vs. Self-Direction, Power, Achievement, Universalism, and Stimulation. The Spearman rank-correlation between the two factor scores amounts to ρ = 0.04, a very small deviation from the product-moment correlation of *r* = 0.00, mainly because of a tie.

**TABLE 5:** Factor score coefficients and the relative importance given to individual level values across countries in Europe.

	Factor score coefficients			
Value type classification	Dimension 1	Rank of importance	Dimension 2	Rank of importance
Conformity	0.15	6	−1.12	2
Tradition	0.51	7	−1.07	3
Security	1.03	10	−0.36	5
Power	0.98	9	1.14	9
Achievement	1.22	11	0.81	7.5
Hedonism	0.87	8	−0.24	11
Stimulation	−0.25	5	0.68	6
Self-Direction	−1.00	2	1.41	10
Universalism	−1.56	1	0.81	7.5
Benevolence	−0.98	3.5	−0.74	4
Not Classified	−0.98	3.5	−1.34	1

*Factor scores are extracted with a PCA with varimax rotation. Correlation between factors is *r* = 0.00. The Spearman rank-correlation between the factor scores is ρ = 0.04.*

Factor *loadings* on the two factors were treated as country coefficients of two distinct dimensions of the latent cultural value profile in Europe. The correlation between factor loadings was *r* = −0.92, which indicated a high degree of similarity across countries in Europe in terms of how well they were represented by the observed common cultural value profile (the factor scores, which were orthogonal). The value profile of each individual country was reproduced by its unique loadings on the European latent value profile. A country that had a (very) high loading on the first factor score had a straightforward value profile that was reliably represented by this factor (e.g., Scandinavian countries and Iceland) and thus required no structure modifications introduced by the second factor. However, for countries with smaller loadings on the first factor (e.g., Russia, Ukraine), the latent value profile summarized by the second factor became increasingly more important. In such cases, the empirical value profile must be adjusted based on the latent value profile of the second factor. To summarize, the very high negative correlation between factor loadings indicates that the value profiles of all countries in Europe (available in the dataset) can be reproduced by two latent cultural value dimensions in a specific way: The dominant value profile in Europe is the profile of the first factor and the deviation from this latent profile is best described by the increasing influence of the modification by the second latent profile, the greater the deviation the higher the loadings of the second factor.

**FIGURE 2:**
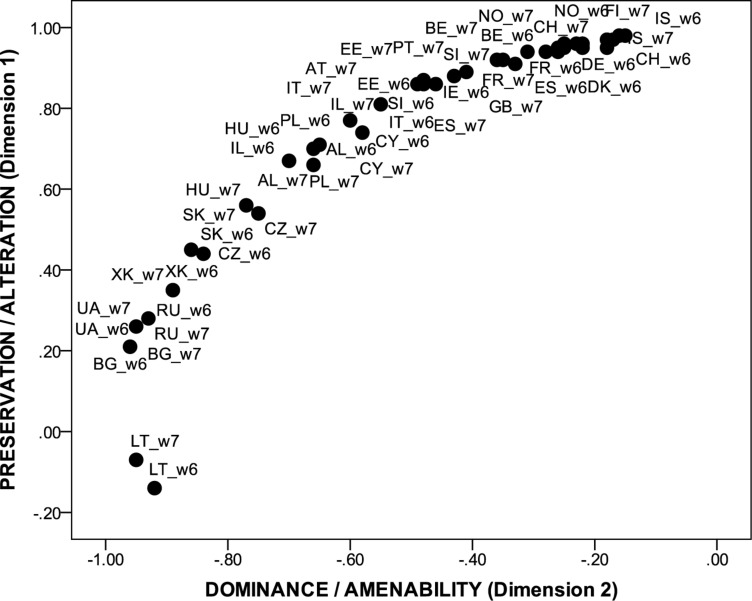
The position of European countries along two dimensions of cultural values as informed by the distribution approach. Preservation/alteration value rank order: (+) universalism, self-direction, benevolence, and stimulation vs. achievement, security, power, hedonism, traditionalism, and conformity (–); dominance/amenability reversed value rank order: (+) self-direction, power, achievement, universalism, and stimulation vs. conformity, traditionalism, benevolence, security, and hedonism (–); 20 countries were available in both rounds of the ESS, namely: Belgium (BE), Switzerland (CH), Czechia (CZ), Germany (DE), Denmark (DK), Estonia (EE), Spain (ES), Finland (FI), France (FR), Great-Britain (GB), Hungary (HU), Israel (IL), Ireland (IE), Lithuania (LT), the Netherlands (NL), Norway (NO), Poland (PL), Portugal (PT), Sweden (SE), and Slovenia (SI), nine countries were available only in Round 6 of the ESS, namely: Albania (AL), Bulgaria (BG), Cyprus (CY), Iceland (IS), Italy (IT), Russian Federation (RU), Slovakia (SK), Ukraine (UA), and Kosovo (XK). One country was only available in Round 7 of the ESS, namely Austria (AT).

Figure 2 shows how countries in the data set were positioned in the two-dimensional space of the latent cultural value profile (country loadings on the two factors) in Europe. Exempting Lithuania, all countries were positioned in the positive sector of Factor 1 and all countries, no exception, were positioned in the negative sector of Factor 2, which was numerically inverted to ease interpretation. There was a clear linear arrangement of countries in the European context. Whereas countries from the former Communist bloc were grouped at the lower ends of the bi-modal space, countries from Central Europe and Western Europe were clustered at the upper ends of this space. Iceland, Switzerland and all Scandinavian countries were situated in the highest echelons.

Next, we sought to identify indicators that can explain the amount of deviation of a country’s profile from the dominant value structure of the first factor. Due to the high negative correlation of the loadings we know that the deviation from the dominant profile is also an increasing similarity with the profile of the second factor. Nevertheless, we will give the results from both analyses. We first correlated the factor loadings with the proposed objective indicators (see the Supplementary Table 2). This was done to identify each indicator’s independent association with the two factor scores (latent cultural dimensions). Then, two multiple hierarchical regressions were conducted (one each for the economic and the socio-political indicators). The per-country factor loadings of the two dimensions were treated as dependent variables and all the objective indicators were treated as predictors. This identified the ‘unique’ variance that was predicted by one indicator after partialling out the effects of other indicators.

Table 6 summarizes the results. Concerning the economic indicators, GDPpc predicted higher country loadings on Dimension 1, *b* = 0.72, *t*(57) = 8.15, *p* < 0.001, and lower country loadings on Dimension 2, *b* = −0.78, *t*(57) = −10.23, *p* < 0.001. In reverse fashion, GINI predicted lower country loadings on Dimension 1, *b* = −0.23, *t*(57) = −2.60, *p* = 0.01, and higher country loadings on Dimension 2, *b* = 0.30, *t*(57) = 3.87, *p* < 0.001. Concerning the socio-political indicators, country loadings on Dimension 1 were predicted by higher life expectancy, *b* = 0.87, *t*(49) = 9.00, *p* < 0.001, lower scores for civil liberties (which stand for *more* civil liberties), *b* = −0.20, *t*(49) = −2.17, *p* = 0.03, and lower percentage of religious people, *b* = −0.19, *t*(49) = −2.45, *p* = 0.02. Furthermore, country loadings on Dimension 2 were predicted by lower life expectancy, *b* = −0.66, *t*(49) = −7.20, *p* < 0.001, higher percentage of religious people, *b* = 0.17, *t*(49) = 2.34, *p* = 0.02, and higher percentage of non-classifiable people, *b* = 0.31, *t*(49) = 3.79, *p* < 0.001. The latent value profile of the first factor was associated with all positive conditions and the latent value profile of the second factor was associated with the negative conditions in a country.

**TABLE 6:** Explanations of the cultural dimensions of the distribution approach.

	Preservation/Alteration (Dimension 1)				Dominance/Amenability (Dimension 2)			
Economic/Social factors	ß	*t*	*p*	95% CI	ß	*t*	*p*	95% CI
GDPpc (rev)	0.72	8.15	< 0.001	0.01; 0.01	−0.78	−10.23	< 0.001	−0.01; −0.01
GINI	−0.23	−2.60	0.01	−0.03; −0.01	0.30	3.87	< 0.001	0.01; 0.03
*Adj. R*^2^	0.54				0.66			
*N*	60				60			
Education	−0.01	−0.12	0.90	−0.04; 0.03	−0.06	−0.86	0.39	−0.04; 0.02
Life Expectancy	0.87	9.00	< 0.001	0.05; 0.08	−0.66	−7.20	< 0.001	−0.06; −0.03
Ethnic Fractionalization	−0.05	−0.65	0.52	−0.33; 0.17	0.04	0.66	0.51	−0.14; 0.29
Civil Liberties (rev)	−0.20	−2.17	0.03	−0.10; −0.01	0.06	0.75	0.46	−0.03; 0.06
% Religious People	−0.19	−2.45	0.02	−0.01; −0.01	0.17	2.34	0.02	0.00; 0.01
% Non-classified People	−0.15	−1.74	0.09	−0.01; 0.01	0.31	3.79	< 0.001	0.01; 0.01
*Adj. R*^2^	0.77				0.79			
*N*	56				56			

Standardized coefficients shown; *n* decreases from 60 to 56 for the regression on social factors because data do not exist for countries Slovakia and Kosovo on ‘Ethnic Fractionalization’ and for Kosovo on ‘Life Expectancy’; (rev) = reversed – higher coefficients pertain to (a) more products and services per capita and (b) more civil liberties; GDPpc = Gross Domestic Product per capita; GINI = inequality index; response anchors for individual values; 1 *– very much like me*, 6 *– not like me at all*; * *p* < 0.05; ** *p* < 0.01, *** *p* < 0.001.

In a final step, we created indices of ‘societal challenges’ to move toward a generalized interpretation of cultural value dimension as informed by the Distribution Approach. We *z*-transformed indicators with a significant regression coefficient (see above) and we created indices by summing them up^1^. Reverse coding was applied to align the objective indicators in terms of low or high coefficients. Two indicators of societal challenges were therefore constructed. Small societal challenges were defined as high GDPpc, low GINI, high life expectancy, high civil liberties and low proportions of religiosity. High societal challenges were defined as low GDPpc, high GINI, low life expectancy, and high proportion of religiosity. Finally, we correlated these indices with the country-loadings on the two dimensions (for a similar procedure see Wainer, 1976). Low societal challenges correlated positively with Dimension 1, *r* = 0.76, *p* < 0.001, and negatively with Dimension 2, *r* = −0.81, *p* < 0.001. High societal challenges correlated negatively with Dimension 1, *r* = −0.78, *p* < 0.001, and positively with Dimension 2, *r* = 0.83, *p* < 0.001. The two societal challenges indices were also correlated with SWB, namely, low societal challenges, *r* = 0.74, *p* < 0.001, high societal challenges, *r* = −0.76, *p* < 0.001. Evidently, the latent cultural value profile of the first factor (Dimension 1) can be found under relatively positive socio-economic conditions in Europe and is by far the predominant one across European countries (explains 60% of the variance). The second latent cultural value profile (Dimension 2) is particularly relevant in contexts with high societal challenges and therefore has the function of modifying the otherwise dominant European latent cultural value profile (explains an additional 30% of the variance).

Finally, the country loadings on the two dimensions were correlated with SWB; Dimension 1, *r* = 0.73, *p* < 0.001, Dimension 2, *r* = −0.81, *p* < 0.001. The complexity of value profiles of countries increases under moderate levels of SWB because the influence of the latent value profile of the second factor becomes more important. Populations with extremely high or low SWB, which corresponds to well-being shaped by high vs. low societal challenges, reproduce a straightforward and simplistic cultural value profile in their countries. For instance, the cultural value profiles of Scandinavian countries are almost perfectly reproduced by the first latent common profile existent in Europe. Conversely, the cultural value profiles of East European countries are almost entirely reproduced by the second latent common profile existent in Europe.

## Discussion

### Similar but Antagonistic Tendencies in the Observed Distribution of Cultural Values

The current paper propagates an alternative and psychometrically sound approach to the assessment of cultural values as suggested by Shalom Schwartz. We have argued that the dominant averaging approach to empirically infer values at the culture level contradicts the theoretical propositions of the circumplex nature of value priorities at the individual level. The averaging approach accommodates insufficiently the at-times-negative and null correlations between value profiles across the 10 value types for individuals. We have suggested a different approach to measuring culture-level value preferences, namely one that we call the distribution approach. Unlike the averaging approach, which looks at average scores of the individual preferences, the distribution approach looks at frequencies of individuals who prefer each of the 10 values *most* in each culture. Moreover, the approach checks to which degree preferences for the other nine values follow the conceptual assumptions of Schwartz’s individual-level circumplex model of value preferences.

This article brings new insights to the culture-level value theory proposed by Schwartz. Value priorities are a characteristic of the individual. Thus, values at the cultural level represent a specific frequency distribution of value priorities of members of the culture. A fictitious middle individual as a prototype for a culture is a problematic approximation both from a conceptual and methodological viewpoint. Culture is an abstract construct that resides outside the individual meaning that the process of averaging across individuals in a culture is–vis-à-vis the assumption of the well-corroborated circumplex model of individual value–an inadmissible procedure. The interpretation of culture-level dimensions based on average scores does not give justice to the theory of value priorities at the individual level and therefore should be avoided in future research.

Our findings suggest two theoretical ways of how individual values extend to values at the culture level (see Table 5). The first is almost an identical reproduction of the circumplex model of individual-level values at the culture level and has little to nothing to do with the cultural dimensions proposed by Schwartz. The second dimension of intercultural values diverges slightly from the first insofar that it does not follow adequately the proposed circumplex model at the individual level.

**FIGURE 3:**
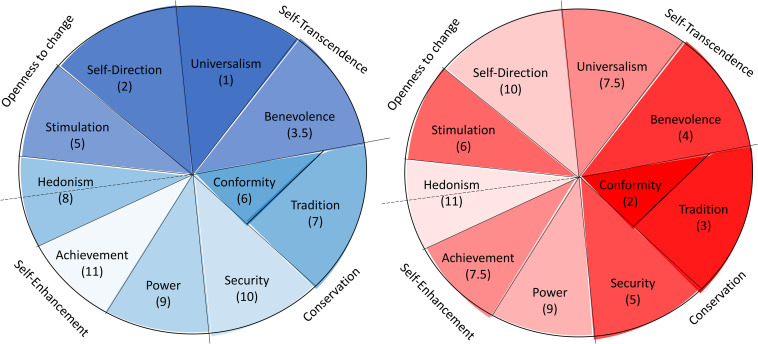
Depiction of the here-conceptualized culture level value dimensions of Preservation/Alteration (blue) and Dominance/Amenability (red). Numbers correspond to value rank order of importance reported in Table 4. The rank of the non-classified has been eliminated. The stronger emphasized colors correspond to value typologies with a higher weight on the respective dimension. The weaker emphasized colors correspond to value typologies with a lower weight on the respective dimension. To arrive at the value structure of a culture, one requires the weights of the respective culture (factor loadings) on the two dimensions.

The present findings show that culture-level values can be structured along two dimensions that describe two widely identified societal tendencies, namely maintaining the *status quo* and progression from the *status quo*. This two-dimensional structure is well supported by empirical evidence (Bilsky, 2008; Strack et al., 2008). Moreover, this is the very manner in which Schwartz has proposed his theory of individual-level values–there is one dimension which describes (the degree of) preservation of the *status quo* in individual values (Conservation vs. Openness to Change) and one dimension which describes self vs. other-focused values (Self-Enhancement vs. Self-Transcendence) (Schwartz, 2012). Our approach shows how these tendencies, which occur at the individual level, can be captured adequately from a measurement standpoint at the cultural level as well. We propose the following nomenclature of cultural values dimensions: *Preservation/Alteration* and *Dominance/Amenability*. This terminology also follows the earlier work by Boehnke, who proposed a slightly different re-conceptualization of Schwartz’s then still nascent theory of cultural values (Boehnke, 1993). A visual guide to the culture level value dimensions as it is informed by the distribution approach can be seen in Figure 3.

### A Theoretical Case for the Two Value Dimensions Informed by the Distribution Approach

We have argued that the value profile of a country is a weighted combination of the two newly identified latent profiles of cultural values. In Europe, there is a clear dominance of the value profile known from research on individual values: Universalism, Self-direction, Benevolence, and Stimulation (*Alteration*) vs. Achievement, Security, Power, Hedonism, Tradition, and Conformity (*Preservation*). This dimension is the core of the European value orientation. However, this orientation in a country requires low levels of societal challenges. With increasing societal challenges, the country’s value orientation is modified. The strength of this modifier (factor loadings) depends on the presence, or lack of, societal challenges: the better the socio-political conditions, the higher the weight on the distribution of the first factor scores. The two dimensions and the sum of the weights is almost equal for all countries, which implies an almost perfect representation of countries’ value profiles along the two value dimensions (over 94% explained variance), with a highly negative correlation of the loadings of the two factors. The high correlation of the loadings does not mean that the distribution of the factor scores on the second factor may be deduced from this correlation. The second factor is not to be interpreted as redundant because of the high negative correlations of the factor loadings. The factor scores of the second factor are independent from the factor scores of the first factor. The specific values of the second factor help us understand their modifying influence against the values of the first factor.

**FIGURE 4:**
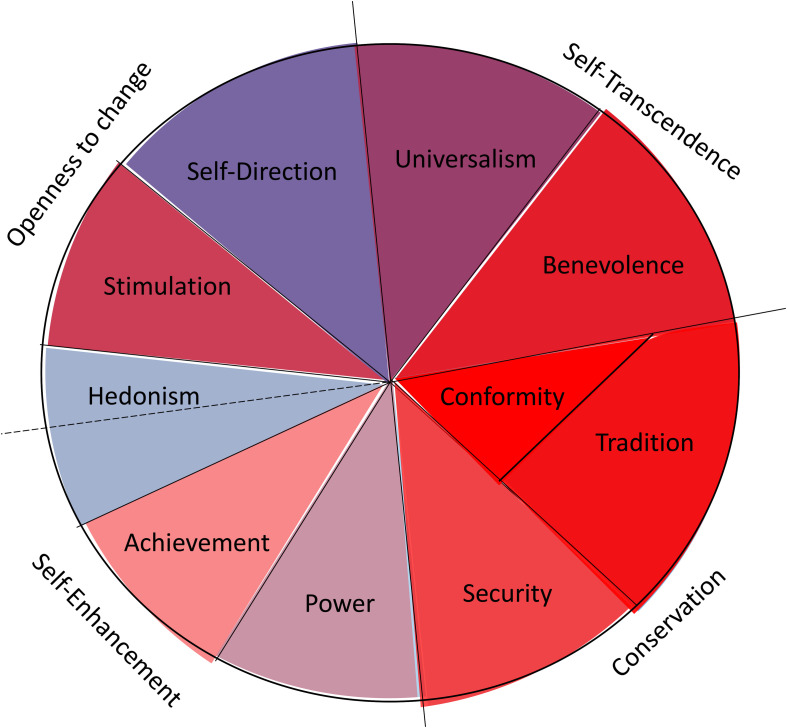
Illustration of the effect the modifier dimension (Dominance/Amenability) has on the value structure in the general European culture. The illustration corresponds to the hypothetical European culture whereby the dimension of Dominance/Amenability perfectly modifies the core dimension of Preservation/Alteration. The core value profile has been changed into the direction of more stability (Traditionalism, Conformity) and Security with individual satisfaction (Hedonism) and less external influence by Stimulation and general connection (Universalism). The individual motivation is less pronounced (Power, Achievement, Self-direction) and the general helping behavior (Benevolence) has remained virtually unmodified.

The second dimension–the modifier–has three functions: (1) it increases the importance of certain values of the first dimension, (2) it decreases the importance of the values in a specific country due to the circumstances based on the European value ideal (for a visual guide see Figure 4) or (3) it does not change the importance of certain values’ rank positions. It is easy to see these three influences in Table 5. Comparing the ranks of the values on the two dimensions the importance of the following values increases (a positive difference of the value ranks of Factor 1 minus Factor 2 greater than 1 or a negative difference greater than 1): Conformity, Tradition, Security, and Achievement are more highly preferred under Dimension 2. Hedonism, Self-Direction and Universalism were found to be less highly preferred under the second dimension, whereas the unchanged values are: Power, Stimulation, and Benevolence, equally (un-)important under both dimensions. That is, by including Dimension 2, the general European value profile becomes more conservative (Tradition, Conformity, and Security) and competitive (Achievement) and less individualist, regarding Hedonism, Self-Direction, and Universalism. Individual activity (Power, and Stimulation) as well as Benevolence are not affected by the two dimensions; their ranks are essentially identical under the two dimensions. Solving the problem of societal challenges, the country concentrates on its *status quo* with high security but less personal satisfaction and a less self-transcendent perspective. The shared image of European citizens is not the self-directed person with internal motivation and satisfaction–individualistic orientation–but a person who is adapted to the tradition and security–a more collectivistic orientation. Due to the economic problems there are also no resources to help other countries. This kind of a more collectivistic orientation will be found in Eastern European countries as we see empirically in our data and in daily observations (see below).

### (Re-)Conceptualization of Cultural Values as Theorized by Schwartz

We have examined to what degree several context variables predicted countries’ relative positioning on the two dimensions of cultural value priorities. Regarding the objective *economic* indicators, findings suggest that the organization of values according to *Preservation/Alteration* is preferred under high levels of economic prosperity and small income inequalities. Conversely, the organization of values according to *Dominance/Amenability* is preferred under conditions of low economic prosperity and large income inequalities. Moreover, as it can be seen from the correlations with subjective well-being, whereas the first cultural value dimension seems to be adopted under perceived low economic stress, the second cultural value dimension seems to be adopted under perceived high economic stress.

With regards to objective *sociopolitical* indicators, high life expectancy is by far the most influential contributing factor to the organization of values according to *Preservation/Alteration*. This is not a surprise given the strong association with economic development. A surprising result is, however, the negative prediction of the order of values of *Preservation/Alteration* by civil liberties. Whereas one could intuitively interpret the simple correlation between the two as evidence that more civil liberties in a country facilitate the prioritization in the country of values such as Universalism, Self-Direction, Benevolence, and Stimulation, the results of the hierarchical regression is less intuitive. An explanation is offered by the way the method calculates the unique prediction of a predictor after partialling out the influence of the other predictors. That is, after the common influence of civil liberties and the economic sector and life expectancy (see intercorrelations) is set aside, only those aspects of civil liberties remain that have to do with values of Achievement, Security, Power, Hedonism, Tradition, and Conformity. Religiosity, on the other hand, intuitively reconstructs the reversed order of values of *Preservation/Alteration*. Short life expectancy and high religiosity are country conditions that can facilitate the organization of values at the culture level according to *Dominance/Amenability*. Overall, the results suggest that the structure of value priorities at the culture level, as informed by the distribution approach, is associated with levels of societal challenges that exists in a country. Correlations between subjective well-being and our newly created indices of societal challenges confirm that individuals report higher satisfaction with life under conditions of low-level societal challenges, and lower satisfaction with life under high societal challenges, which is no surprise only a validation of the indices.

This initial evidence posits that when societal challenges are perceived as worries (see correlations with SWB) (Boehnke et al., 1998), priority is given to specific values that correspond mostly to goals of individuals. If we are to use as an analogy Maslow’s pyramid of basic needs (Maslow, 1943), and parallel existing theories of human development (e.g., Welzel et al., 2003), then it is clear that under worries individuals would not have the necessary human condition to seek values which would allow their concern with aspects of transcendence. In other words, it seems that the here proposed structure of values at the culture level reproduces under societal challenges those values that facilitate the basic survival nature of individuals, e.g., *Preservation* and *Dominance* (Witte and Zenker, 2016). In contrast, it appears that under low worries those values are reproduced that facilitate the transcending nature of individuals, e.g., *Alteration* and *Amenability*. Further evidence for this interpretation is given by the proportion of non-classified individuals into one of the 10 basic value types–proportion of people in a country with an arbitrarily deviating value structure is associated with increased chances that in the country values are organized according to *Preservation* and *Dominance*.

### What Can We Learn From the Non-classified Individuals?

About 30 % of the cases in the data set could not be classified in our approach into one of the 10 value types theorized by Shalom Schwartz. Golan and Witte reported identical results based on Rounds 3 (2006) and 4 (2008) of the ESS data (Gollan and Witte, 2014). Therefore, across four data sets (ESS rounds in 2006, 2008, 2012, and 2014) that had been collected across more than 35 countries, contain information from approximately 147,000 individuals, and are over 8 years apart, one conclusion may be drawn: The value preferences of a large proportion of individuals in European countries (Turkey and Israel included) do not organize as the theory of individual level value preferences would predict. One interpretation is that the value preferences of these individuals organize in a way that differs from the proposed compatibility-incompatibility relationship amongst them. Please note that we do *not* consider disregarding the compatibility-incompatibility structure, rather we are proposing that, whereas this structure likely still holds, the values that should theoretically be in contradiction with one another may in fact be similarly prioritized by the non-classified individuals. For example, one might expect that classified individuals attribute similar priorities to Universalism and Power. In contrast, there is a possibility that non-classified individuals attribute converging priorities to Universalism and Power.

The classification of individuals into one of the 10 value types is based on rather a lenient criterion. As Borg and colleagues argue, the criterion (*r* ≥ 0.50) only allows for the unfolding of the values with the highest and the lowest priorities of the circumplex model (Borg et al., 2011); the priorities of all the values in-between are by and large unknown. The analogy to psychometric scale development could once again be insightful here. It is argued that the higher the internal consistency of a set of items the more reliable a scale is toward the measurement of a given psychological construct. Likewise, should the criterion for the classification of individuals into one of the 10 value types be more conservative (for example, *r* ≥ 0.80), then the circumplex model of cultural values will be more adequately applied empirically. It is likely then, however, that a high proportion of individuals cannot be classified into one of the 10 original value types, which would be an intriguing finding in and by itself, because of the reproduction of this two-dimensional structure in countless studies.

### Limitations and Future Directions

There are limitations to the generalizability of our findings. First, the research focuses on data available from European countries (includes Turkey and Israel) only. This, however, is not fully representative for the multitude of cultures around the globe. Cultures in Europe are individualistic in general–individuals gain a sense of well-being from personal goals over goals of their groups (Triandis, 1989). Compared to the European context, East-Asian cultures are known to be collectivistic overall–individuals gain a sense of well-being from goals of their groups over their personal goals. It is, therefore, unclear whether the here-proposed structure of cultural values is specific to individualistic cultures alone. The reliability of findings beyond the European context should thus be examined with more comprehensive data, one that includes both individualistic and collectivistic cultures, e.g., by applying the distribution approach to re-examinations of data from the World Value Survey (WVS), which encompasses a one-item-each instrument to assess Schwartz value preferences in its more recent waves.

Second, analyses here are based on the well-known theory of basic human values (10 ideal value types) and not the revised theory which argues for 19 basic human value-types (Schwartz et al., 2012). Whereas there is a pragmatic reason for the present choice of data (unavailability of large cross-cultural, representative data sets for the refined values compared to the original values), there remains a possibility that the distribution approach exhibits a different structure of cultural values when the refined theory of basic values cultural values is used as the ideal structure of values at the individual level. The present procedure should thus also be applied to the data of Schwartz et al. (2012) to examine this possibility.

Overall, the findings represent an attempt toward a sound method to assessing Schwartz’s theory of cultural values. We have argued that the *status quo* in the literature–the averaging approach–reproduces inadequately the theory of culture level value priorities in empirical endeavors (also see the Supplementary, “Logic of the Averaging Approach and the Distribution Approach compared”). We have shown empirical evidence in support of the distribution approach as a methodology based on a meaningful measurement procedure that future research on culture-level values should use. Cultural values in Europe are almost perfectly described by two independent latent value profiles that operate together. Under specific societal challenges, a country has developed its own specific culture level value priorities. We should, finally, admit that the chances that the distribution approach gains major ground in determining culture-level value preferences is not overly high, as the proposed calculation is much more cumbersome than working with the averaging approach. Nevertheless, we believe that the higher cross-level validity of the distribution approach is worth ‘the extra mile’ and this approach is the direct theoretical extension of the value theory for individuals to a value theory of cultures. This is a first attempt toward such a theoretical development at the intersection of psychological and sociological paradigms.

## Data Availability Statement

Publicly available datasets were analyzed in this study. This data can be found here: https://osf.io/r7w2f/.

## Author Contributions

EW contributed study conceptualization, methodology, supervision, writing, and revisions of the original manuscript. AS contributed study conceptualization, data curation, formal analysis, methodology, project administration, validation, visualization, and writing and revisions of the original manuscript. KB contributed resources, and writing and revisions of the original manuscript. All authors contributed to the article and approved the submitted version.

## Conflict of Interest

The authors declare that the research was conducted in the absence of any commercial or financial relationships that could be construed as a potential conflict of interest.

## Footnotes

1 As can be seen from the positive correlations between these indicators such a measurement is meaningful and not an arbitrary index.
