# Supplementary material for: A New Empirical Approach to Intercultural Comparisons of Value Preferences Based on Schwartz’s Theory
Source: Front Psychol. 2020 Jul 14;11:1723. doi: 10.3389/fpsyg.2020.01723 (PMC7371987; doi:10.3389/fpsyg.2020.01723)
Supplement: Supplementary file 1 [file Table_1.DOCX]

Supplementary Material

**Table 1. Country Loadings on the two Dimensions of Cultural Values as Informed by the Distribution Approach.**

|  |  | Factor Loadings | |
| --- | --- | --- | --- |
| Country  Code | Round  ESS | Dimension 1 | Dimension 2 |
| AL | 6 | .66 | .66 |
|  | 7 | .66 | .66 |
| AT | 6 | .86 | .48 |
|  | 7 | .86 | .48 |
| BE | 6 | .95 | .25 |
|  | 7 | .92 | .36 |
| BG | 6 | .21 | .96 |
|  | 7 | .21 | .96 |
| CH | 6 | .96 | .23 |
|  | 7 | .96 | .25 |
| CY | 6 | .74 | .58 |
|  | 7 | .74 | .58 |
| CZ | 6 | .44 | .84 |
|  | 7 | .54 | .75 |
| DE | 6 | .95 | .25 |
|  | 7 | .94 | .26 |
| **DK** | **6** | **.95** | **.22** |
|  | **7** | **.95** | **.25** |
| EE | 6 | .86 | .49 |
|  | 7 | .87 | .48 |
| ES | 6 | .91 | .33 |
|  | 7 | .86 | .46 |
| **FI** | **6** | **.97** | **.17** |
|  | **7** | **.97** | **.18** |
| FR | 6 | .94 | .31 |
|  | 7 | .92 | .35 |
| GB | 6 | .86 | .48 |
|  | 7 | .89 | .41 |
| HU | 6 | .71 | .65 |
|  | 7 | .56 | .77 |
| IE | 6 | .88 | .43 |
|  | 7 | .86 | .49 |
| IL | 6 | .67 | .70 |
|  | 7 | .81 | .55 |
| IS | 6 | .98 | .16 |
|  | 7 | .98 | .16 |
| IT | 6 | .81 | .55 |
|  | 7 | .81 | .55 |
| *LT* | *6* | *-.14* | *.92* |
|  | *7* | *-.07* | *.95* |
| NL | 6 | .95 | .18 |
|  | 7 | .95 | .26 |
| **NO** | **6** | **.96** | **.22** |
|  | **7** | **.94** | **.28** |
| PL | 6 | .77 | .60 |
|  | 7 | .70 | .66 |
| PT | 6 | .70 | .66 |
|  | 7 | .89 | .41 |
| *RU* | *6* | *.28* | *.93* |
|  | *7* | *.28* | *.93* |
| **SE** | **6** | **.97** | **.18** |
|  | **7** | **.98** | **.15** |
| SI | 6 | .81 | .55 |
|  | 7 | .89 | .41 |
| SK | 6 | .45 | .86 |
|  | 7 | .45 | .86 |
| *UA* | *6* | *.26* | *.95* |
|  | *7* | *.26* | *.95* |
| *XK* | *6* | *.35* | *.89* |
|  | *7* | *.35* | *.89* |

AL = Albania, AT = Austria, BE = Belgium, BG = Bulgaria, CH = Switzerland, CY = Cyprus, CZ = Czech Republic, DE = Germany, DK = Denmark, EE = Estonia, ES = Spain, FI = Finland, FR = France, GB = United Kingdom, HU = Hungary, IE = Ireland, IL = Israel, IS = Iceland, IT = Italy, LT = Lithuania, NL = the Netherlands, NO = Norway, PL = Poland, PT = Portugal, RU = Russian Federation, SE = Sweden, SI = Slovenia, SK = Slovakia, UA = Ukraine, XK = Kosovo, **bold emphasized** = country prototypes with a strong emphasis on cultural value dimension 1, *italic emphasized* = country prototypes with a strong emphasis on cultural value dimension 2, underline emphasized = country prototypes with similar emphasis on both cultural dimensions.
