# Supplementary material for: A New Empirical Approach to Intercultural Comparisons of Value Preferences Based on Schwartz’s Theory
Source: Front Psychol. 2020 Jul 14;11:1723. doi: 10.3389/fpsyg.2020.01723 (PMC7371987; doi:10.3389/fpsyg.2020.01723)
Supplement: Supplementary file 2 [file Table_2.DOCX]

Supplementary Material

**Table 2. Inter-Correlations Between the Study Factors.**

|  | Factor | 1 | 2 | 3 | 4 | 5 | 6 | 7 | 8 | 9 | 10 |
| --- | --- | --- | --- | --- | --- | --- | --- | --- | --- | --- | --- |
| 1 | Dimension 1 | 1 |  |  |  |  |  |  |  |  |  |
| 2 | Dimension 2 | -.92*** | 1 |  |  |  |  |  |  |  |  |
| 3 | GDPpc (rev) | .71*** | -.76*** | 1 |  |  |  |  |  |  |  |
| 4 | GINI | -.19 | .25* | .05 | 1 |  |  |  |  |  |  |
| 5 | Education | .26* | -.34** | .46*** | -.16 | 1 |  |  |  |  |  |
| 6 | Life Expectancy | .84*** | -.81*** | .79*** | -.11 | .10 | 1 |  |  |  |  |
| 7 | Ethnic Fractionalization | -.33* | .32** | -.34*** | .11 | -.07 | -.32* | 1 |  |  |  |
| 8 | Civil Liberties (rev) | .51*** | -.56*** | .69*** | -.27* | .25 | .66*** | -.13 | 1 |  |  |
| 9 | % Religious People | -.40** | .46*** | -.37** | .42** | -.41** | -.14 | -.03 | -.24 | 1 |  |
| 10 | % Non-Classified People | -.55*** | .67*** | -.52*** | .33** | -.44*** | -.44** | .24 | -.46*** | .47*** | 1 |

GDPpc = Gross Domestic Product per capita, GINI = inequality index, response anchors for individual values, (rev) = reversed – higher coefficients pertain to (a) more products and services per capita and (b) more civil liberties, scale anchors for value measure, 1 *– very much like me*, 6 *– not like me at all*, *, *p* < .05, **, *p* < .01, ***, *p* < .001.
