# Supplementary material for: A New Empirical Approach to Intercultural Comparisons of Value Preferences Based on Schwartz’s Theory
Source: Front Psychol. 2020 Jul 14;11:1723. doi: 10.3389/fpsyg.2020.01723 (PMC7371987; doi:10.3389/fpsyg.2020.01723)
Supplement: Supplementary file 3 [file Table_3.DOCX]

**Logic of the Averaging Approach and the Distribution Approach compared**

The traditional statistical paradigm postulates that many data, if sufficiently collected, will have a normally distributed structure (the so-called normal curve) which will be described in terms of two parameters: The central tendency (the mean) and the deviation from the central tendency (the standard deviation). By averaging the elements forming the normal curve, we can estimate its mean and the specific confidence interval within which the mean can be found by the standard deviation.

There are situations in which the data are described not by one but by two independent normal curves. In these cases, the average, as a central tendency parameter, is an improper estimation of either of the two normal curves. The estimation is meaningless here because identical central tendency parameters can be derived from nearly infinite combinations of elements of both normal curves. In plain terms, the mean score is an invalid parameter of the data that is known to have more than one normal curve. Because of these hypothetical possibilities, we must examine in practice the internal consistency of data elements: Only when the data have a sufficient internal consistency (for example, as indicated by Cronbach’s *α*) can we be certain that averaging provides valid data parameters.

Considering the case of value research, we know from extensive empirical and theoretical work that the value profiles of individuals in a country are bi-dimensional. Based on the logic explained above, which follows general measurement theory, the average score of the value profiles is meaningless in substantive terms. Therefore, central tendency parameters (the average score in this case) is an invalid approach to operationalizing cultural value typologies.

Our proposition is to describe the cultural value typologies instead by the distribution of the bi-dimensional individual value profiles in a country. We can specify from theory a theoretical distribution of individual value profiles and we can think of these as ideal value typologies. The value profile of a country (culture-level value typology) can be summarized as percentages of individuals in the country whose value profiles have been classified into the ideal value typologies. Similarities between the value profiles of two countries can thus be operationalized as similarities in terms of percentages of individuals whose value profiles match the ideal value profiles in the two-dimensional space. In such countries, there is a similar *distribution* of value types.

Furthermore, one must be aware of the measurement errors that accompany the measurement of individual value profiles, the procedure of classifying individuals’ value profile according to the theorized ideal value typologies, and the biases produced by sampling peculiarities. Because of such measurement errors, we transform percentages of individual value types in a country into the ranks of their percentages which allow for more reliable cross-cultural comparisons.

Table 1. Correlation matrix of the four ideal value profiles (a, b, c, d) in a two-dimensional space of values.

| Value profiles | *a* | *b* | *c* | *d* |
| --- | --- | --- | --- | --- |
| *a* | 1 | -1 | 0 | 0 |
| *b* |  | 1 | 0 | 0 |
| *c* |  |  | 1 | -1 |
| *d* |  |  |  | 1 |

Let us assume two countries, *A* and *B*, with four subgroups each and using independent different value dimensions in constructing their cultural value profile. Groups a and b use primarily the first dimension, Preservation/Alteration, and groups c and d use primarily the second dimension, Dominance/Amenability. Table 1 presents the assumed correlation matrix between the value profiles in general.

The averaging approach uses mean scores across all individuals in computing culture-level indicators of values without differentiating between the underlying two dimensions. For example, in a study where each value typology is measured using a single item on a Likert scale with 4 response options, where 1.0 is *value least important* and 4.0 is *value most important*, one first computes the scores for each individual in the culture. Then, based on these scores, one computes the mean scores for each of the four value profiles but ignores the fact that subgroups use different criteria to build their value profile.

Let us assume further the following ideal mean-based value profiles of the four groups in both countries (see Table 2).

Table 2. The ideal value profile of four subgroups in two different countries.

|  | Group  (% of total population) | Preservation/Alteration | Dominance/Amenability | Average Value Profile |
| --- | --- | --- | --- | --- |
| Country A | a (48%) | 1.0, 2.0, 3.0, 4.0 |  | 2.47, 2.49, 2.48, 2.44 |
|  | b (2%) | 4.0, 3.0, 2.0, 1.0 |  |  |
|  | c (47%) |  | 4.0, 3.0, 2.0, 1.0 |  |
|  | d (3%) |  | 1.0, 2.0, 3.0, 4.0 |  |
| Country B | a (2%) | 1.0, 2.0, 3.0, 4.0 |  | 2.53, 2.52, 2.49, 2.47 |
|  | b (48%) | 4.0, 3.0, 2.0, 1.0 |  |  |
|  | c (3%) |  | 4.0, 3.0, 2.0, 1.0 |  |
|  | d (47%) |  | 1.0, 2.0, 3.0, 4.0 |  |

Considering the average value profile, a comparison between Country A and Country B amounts to almost no differences in terms of cultural value preferences. Thus, rather than using mean scores as a basis for culture-level analyses, we quantify the percentage of the four different subgroups in a culture (see manuscript, Method and Results). We first rank the subgroups in each country according to the percentage they represent in the country. The value rank order in Country A is: a, c, d, b. The value rank order in Country B is: b, d, c, a; this is a total inversion of the second rank order compared with the first. The rank order correlation between the two countries equals to ρ = -1.0. Therefore, a comparison between Country A and Country B would amount to being maximally dissimilar.

To conclude, in a two-dimensional space of value-based profiles, the value profile of the average individual in a country is misleading as a measure of the country’s value profile. Almost identical cross-cultural average value profiles can be informed by highly diverse individual profiles. The averaging approach discards such a diversity (i.e., cultural value profiles would look equal) but not the distribution approach.
